# Supplementary material for: C5aR2 Regulates STING-Mediated Interferon Beta Production in Human Macrophages
Source: Cells. 2023 Nov 25;12(23):2707. doi: 10.3390/cells12232707 (PMC10706378; doi:10.3390/cells12232707)

Figure S1

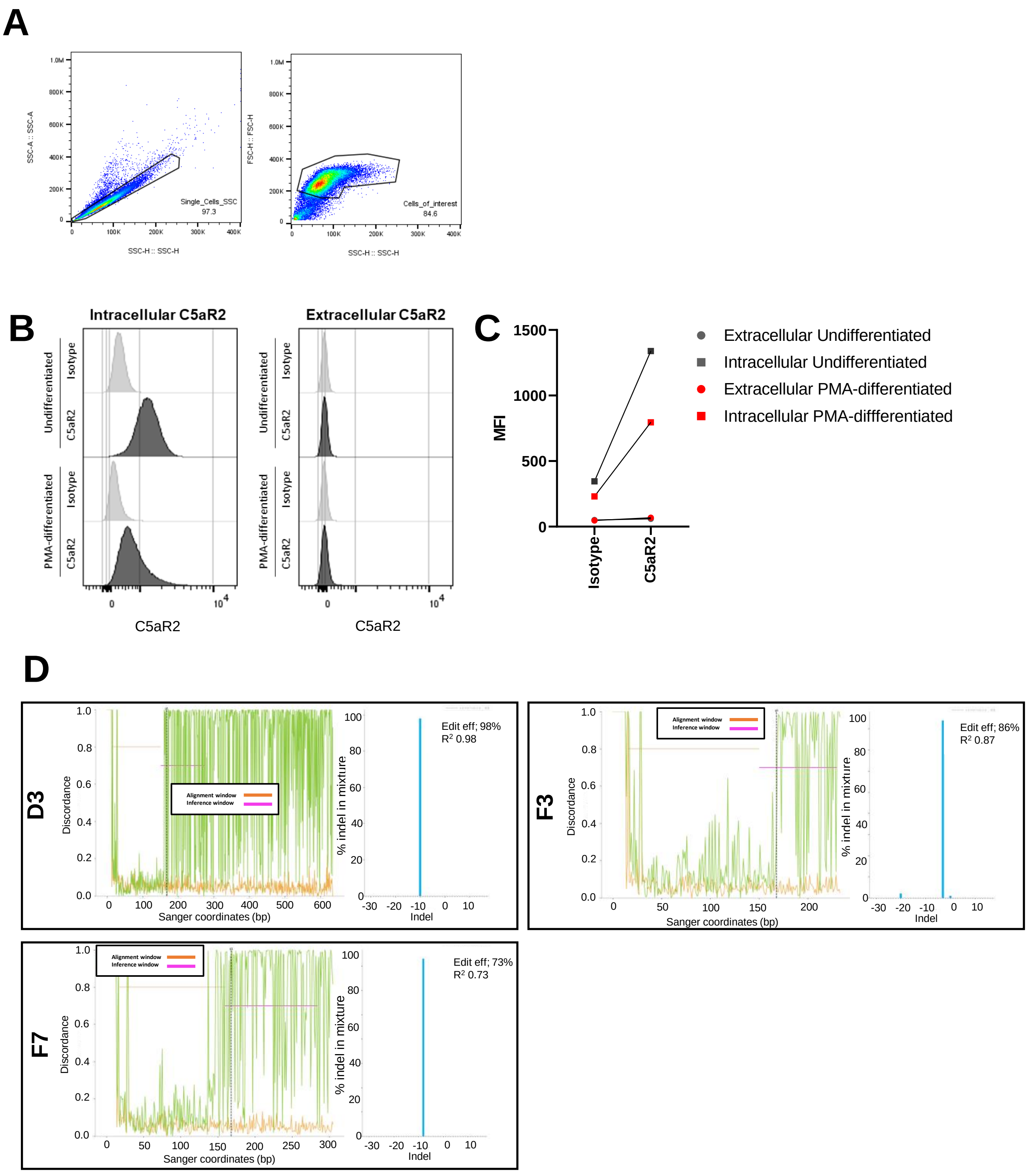
A
Single_Cells_SSC
97.3
Cells_of_interest
84.6
SSC-A :: SSC-A
SSC-H :: SSC-H
FSC-H :: FSC-H
B
Intracellular C5aR2
Extracellular C5aR2
Undifferentiated
PMA-differentiated
Isotype
C5aR2
C5aR2
C
MFI
1500
1000
500
0
Isotype
C5aR2
Extracellular Undifferentiated
Intracellular Undifferentiated
Extracellular PMA-differentiated
Intracellular PMA-diffferentiated
D
D3
F3
F7
Discordance
Sanger coordinates (bp)
Alignment window
Inference window
% indel in mixture
Indel
Edit eff; 98%
R² 0.98
Edit eff; 86%
R² 0.87
Edit eff; 73%
R² 0.73

Figure S2

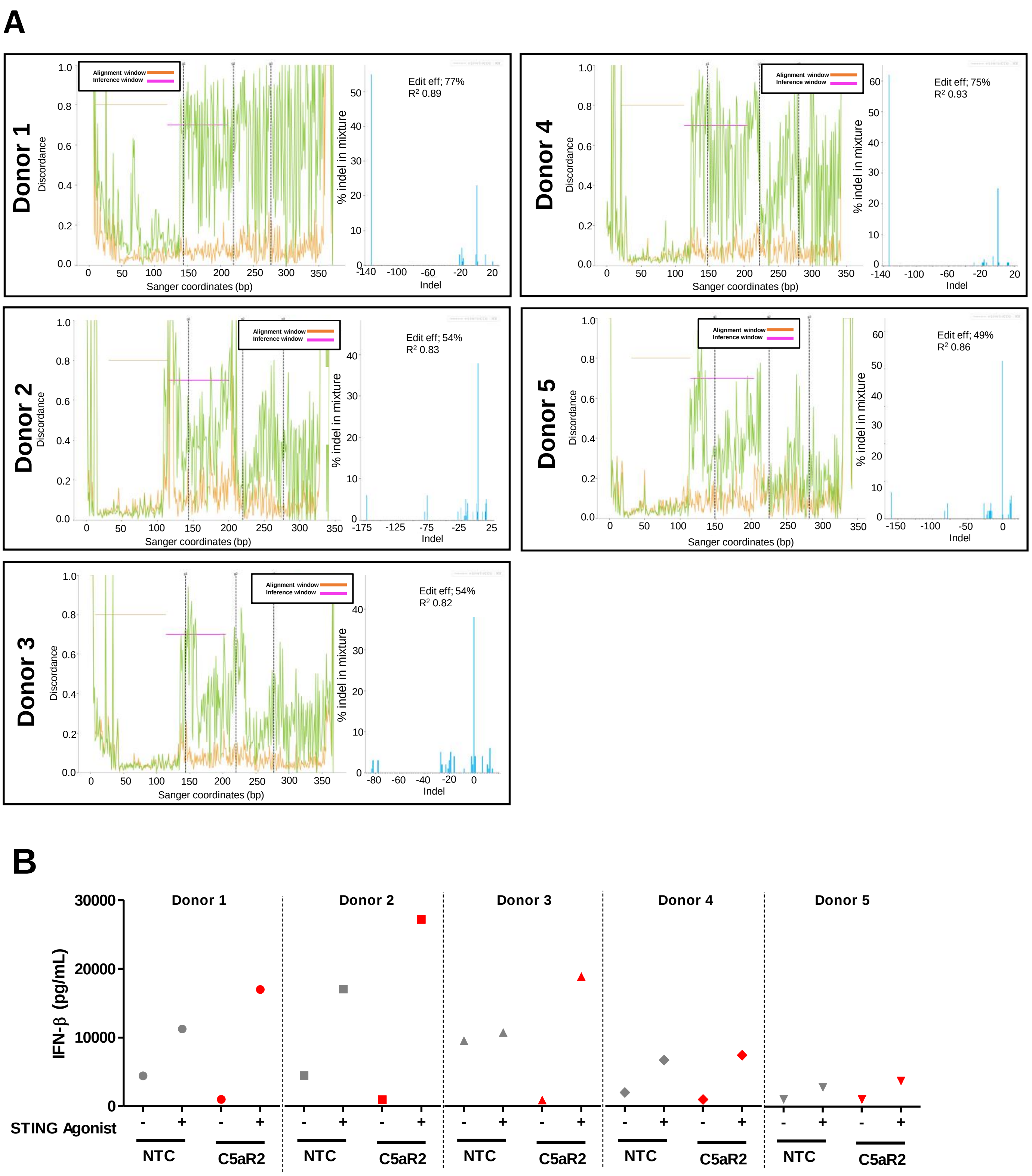
A
Donor 1
Discordance
Alignment window
Inference window
Sanger coordinates (bp)
% indel in mixture
Edit eff; 77%
R² 0.89
Indel
Donor 4
Edit eff; 75%
R² 0.93
Donor 2
Edit eff; 54%
R² 0.83
Donor 5
Edit eff; 49%
R² 0.86
Donor 3
Edit eff; 54%
R² 0.82
B
IFN-β (pg/mL)
Donor 1
Donor 2
Donor 3
Donor 4
Donor 5
STING Agonist
NTC
C5aR2

Figure S3

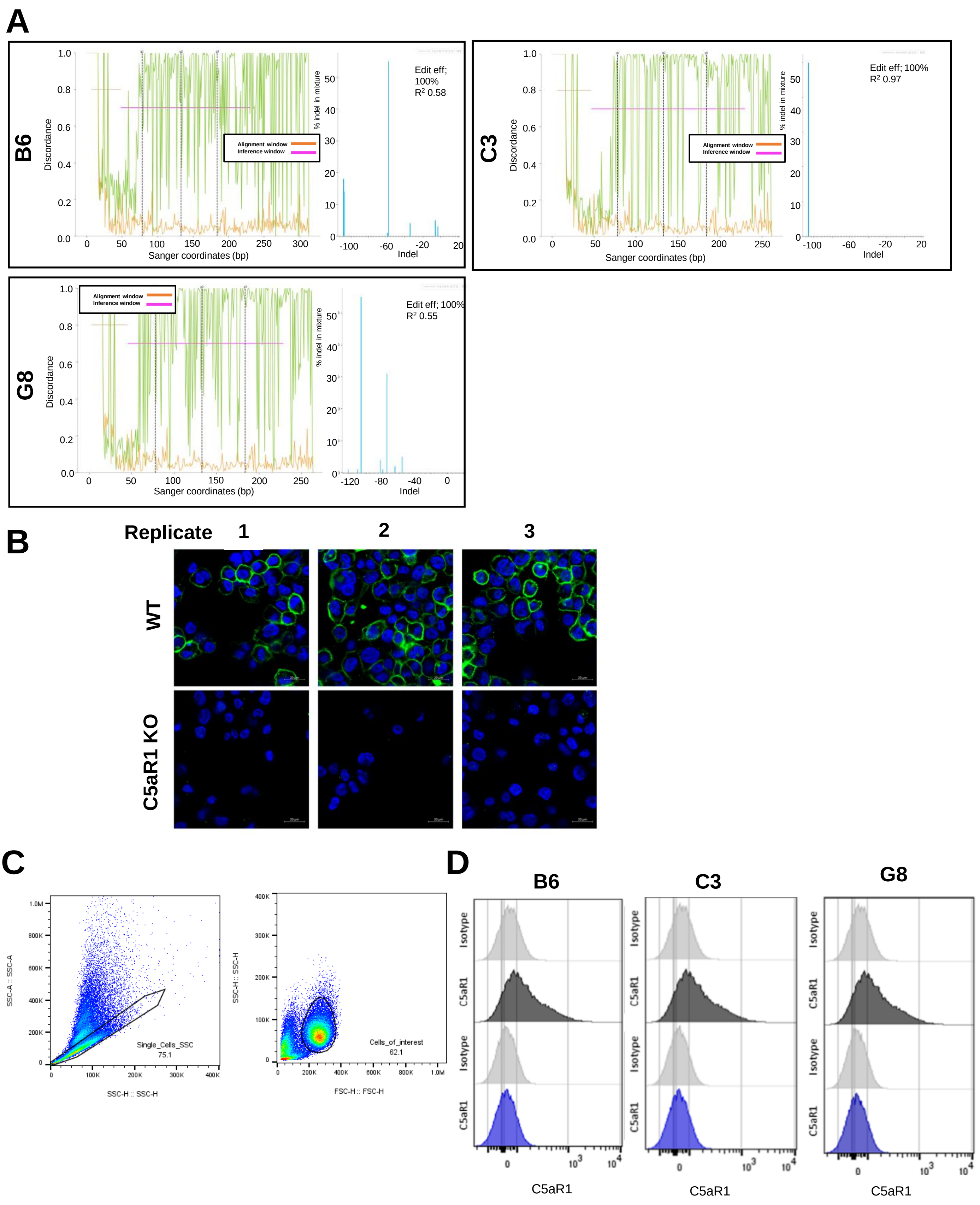
A
B6
C3
G8
Discordance
Sanger coordinates (bp)
Alignment window
Inference window
% indel in mixture
Indel
Edit eff; 100%
R² 0.58
Edit eff; 100%
R² 0.97
Edit eff; 100%
R² 0.55
B
Replicate
1
2
3
WT
C5aR1 KO
C
SSC-A :: SSC-A
SSC-H :: SSC-H
Single_Cells_SSC
75.1
FSC-H :: FSC-H
Cells_of_interest
62.1
D
B6
C3
G8
Isotype
C5aR1
C5aR1

Figure S4

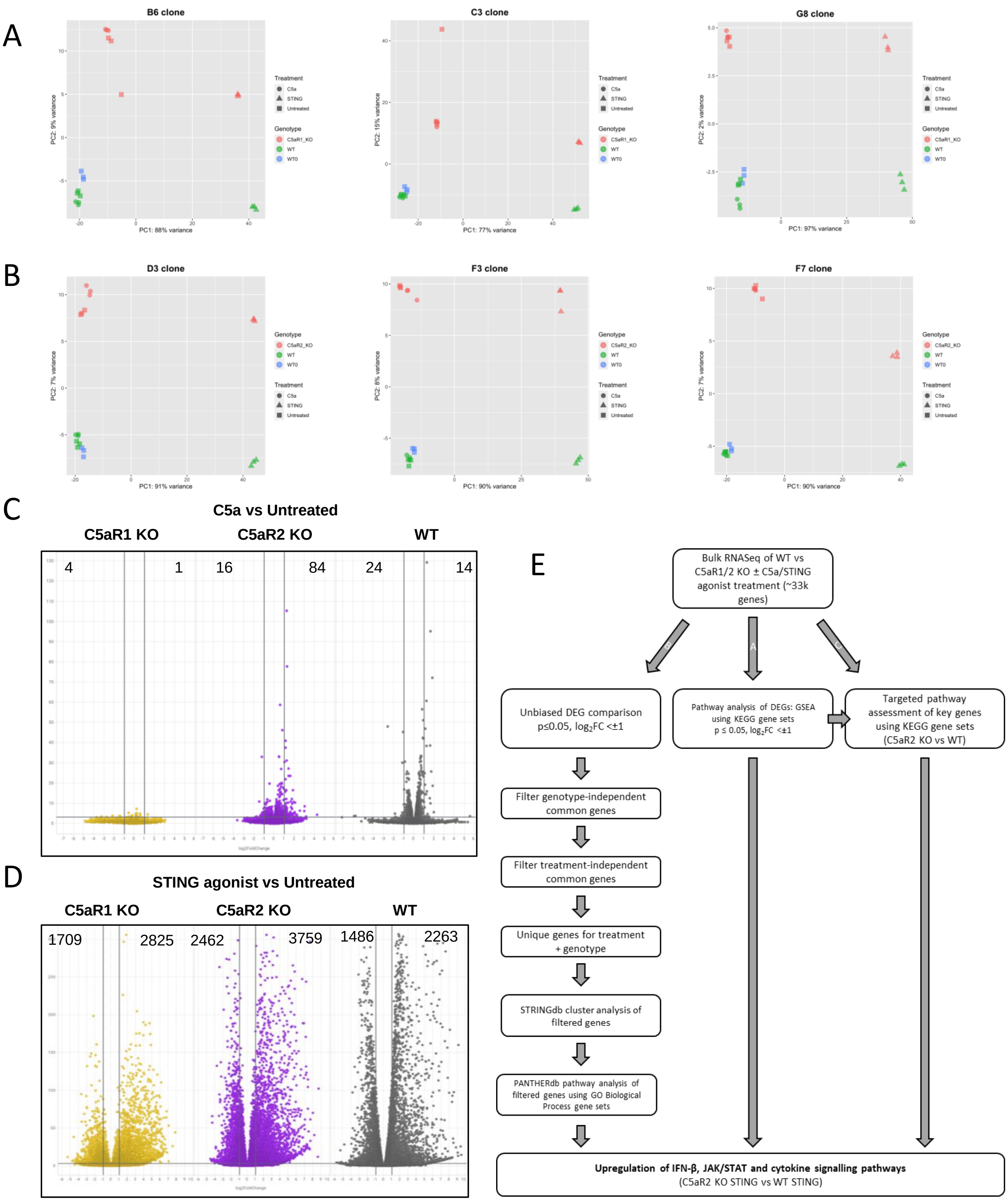
A
B6 clone
C3 clone
G8 clone
PC1: 88% variance
PC2: 9% variance
PC1: 77% variance
PC2: 15% variance
PC1: 97% variance
PC2: 2% variance
Treatment
C5a
STING
Untreated
Genotype
C5aR1_KO
WT
WT0
B
D3 clone
F3 clone
F7 clone
PC1: 91% variance
PC2: 7% variance
PC1: 90% variance
PC2: 8% variance
PC1: 90% variance
PC2: 7% variance
Genotype
C5aR2_KO
WT
WT0
Treatment
C5a
STING
Untreated
C
C5a vs Untreated
C5aR1 KO
C5aR2 KO
WT
4
1
16
84
24
14
D
STING agonist vs Untreated
C5aR1 KO
C5aR2 KO
WT
1709
2825
2462
3759
1486
2263
E
Bulk RNASeq of WT vs C5aR1/2 KO ± C5a/STING agonist treatment (~33k genes)
B
A
C
Unbiased DEG comparison p≤0.05, log2FC <±1
Pathway analysis of DEGs: GSEA using KEGG gene sets p ≤ 0.05, log2FC <±1
Targeted pathway assessment of key genes using KEGG gene sets (C5aR2 KO vs WT)
Filter genotype-independent common genes
Filter treatment-independent common genes
Unique genes for treatment + genotype
STRINGdb cluster analysis of filtered genes
PANTHERdb pathway analysis of filtered genes using GO Biological Process gene sets
Upregulation of IFN-β, JAK/STAT and cytokine signalling pathways
(C5aR2 KO STING vs WT STING)

Fig S5

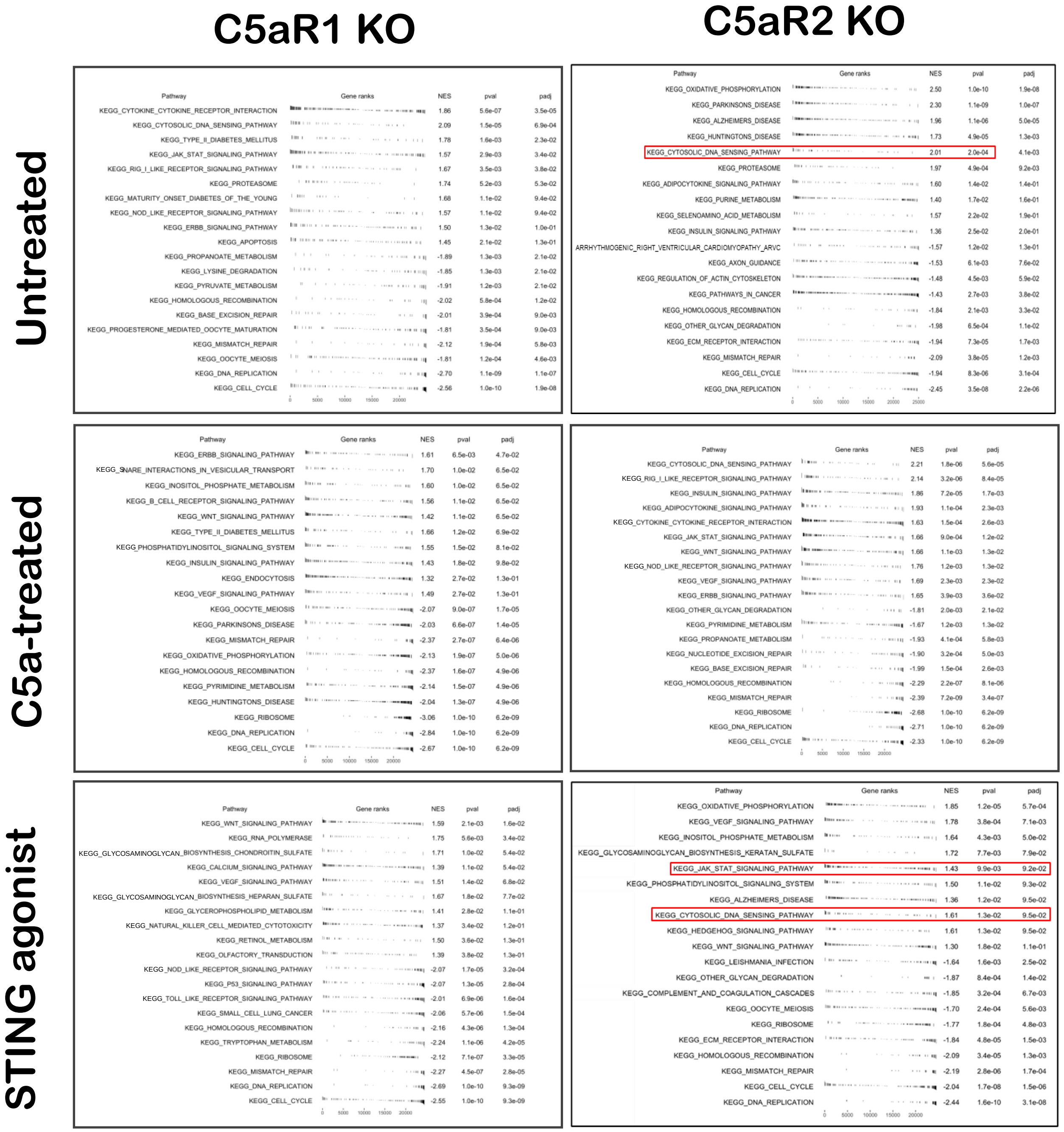

GSEA of wildtype versus C5aR1 (left) or C5aR2 (right) cells, left untreated or treated with C5a or STING agonist.

Figure S6

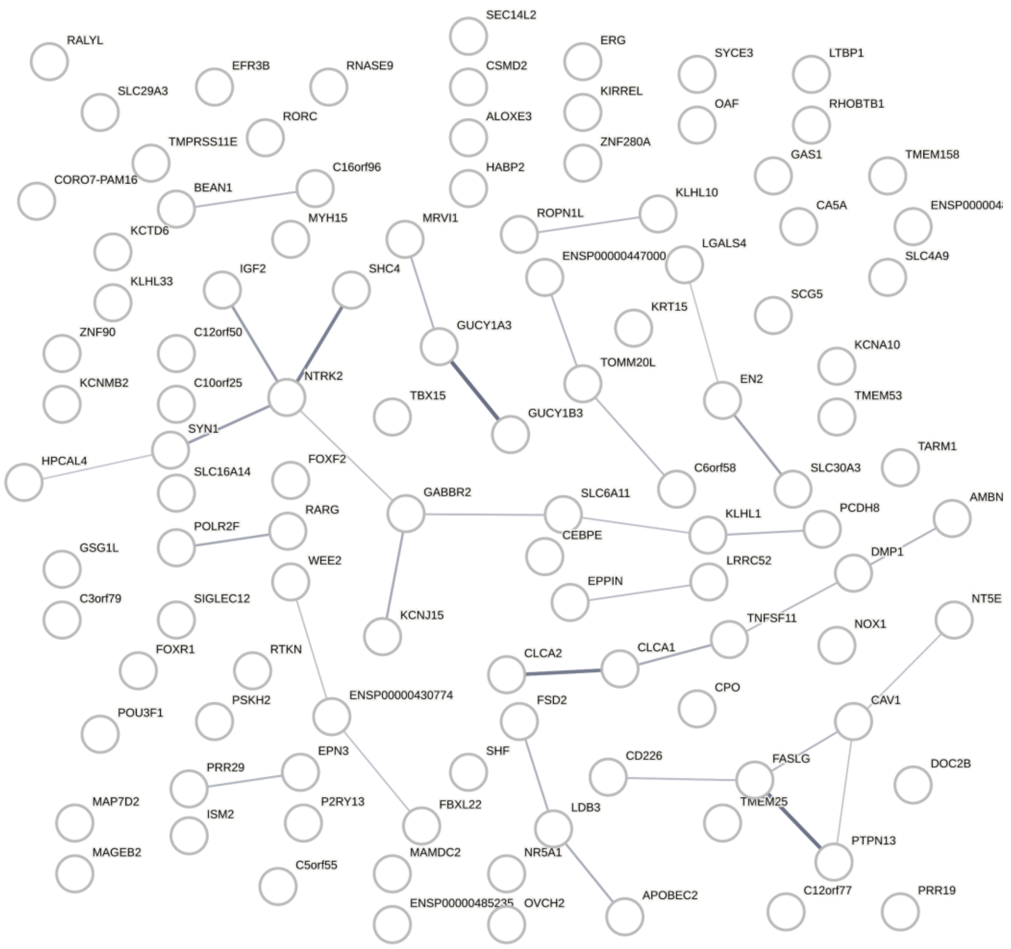
SEC14L2
RALYL
ERG
SYCE3
LTBP1
EFR3B
RNASE9
CSMD2
SLC29A3
KIRREL
RORC
ALOXE3
OAF
RHOBTB1
TMPRSS11E
ZNF280A
GAS1
TMEM158
C16orf96
HABP2
CORO7-PAM16
BEAN1
KLHL10
ROPN1L
CA5A
ENSP000004
MYH15
MRVI1
KCTD6
LGALS4
ENSP00000447000
SLC4A9
IGF2
SHC4
KLHL33
SCG5
KRT15
GUCY1A3
ZNF90
C12orf50
KCNA10
TOMM20L
KCNMB2
C10orf25
NTRK2
EN2
TBX15
TMEM53
GUCY1B3
SYN1
TARM1
FOXF2
HPCAL4
SLC16A14
C6orf58
SLC30A3
GABBR2
SLC6A11
AMBN
RARG
KLHL1
PCDH8
POLR2F
CEBPE
GSG1L
DMP1
WEE2
LRRC52
EPPIN
C3orf79
SIGLEC12
NT5E
TNFSF11
KCNJ15
NOX1
FOXR1
RTKN
CLCA1
CLCA2
CPO
ENSP00000430774
PSKH2
FSD2
CAV1
POU3F1
EPN3
SHF
CD226
FASLG
DOC2B
PRR29
MAP7D2
P2RY13
FBXL22
TMEM25
ISM2
LDB3
PTPN13
MAGEB2
MAMDC2
NR5A1
C5orf55
APOBEC2
C12orf77
PRR19
ENSP00000485235
OVCH2

Figure S7

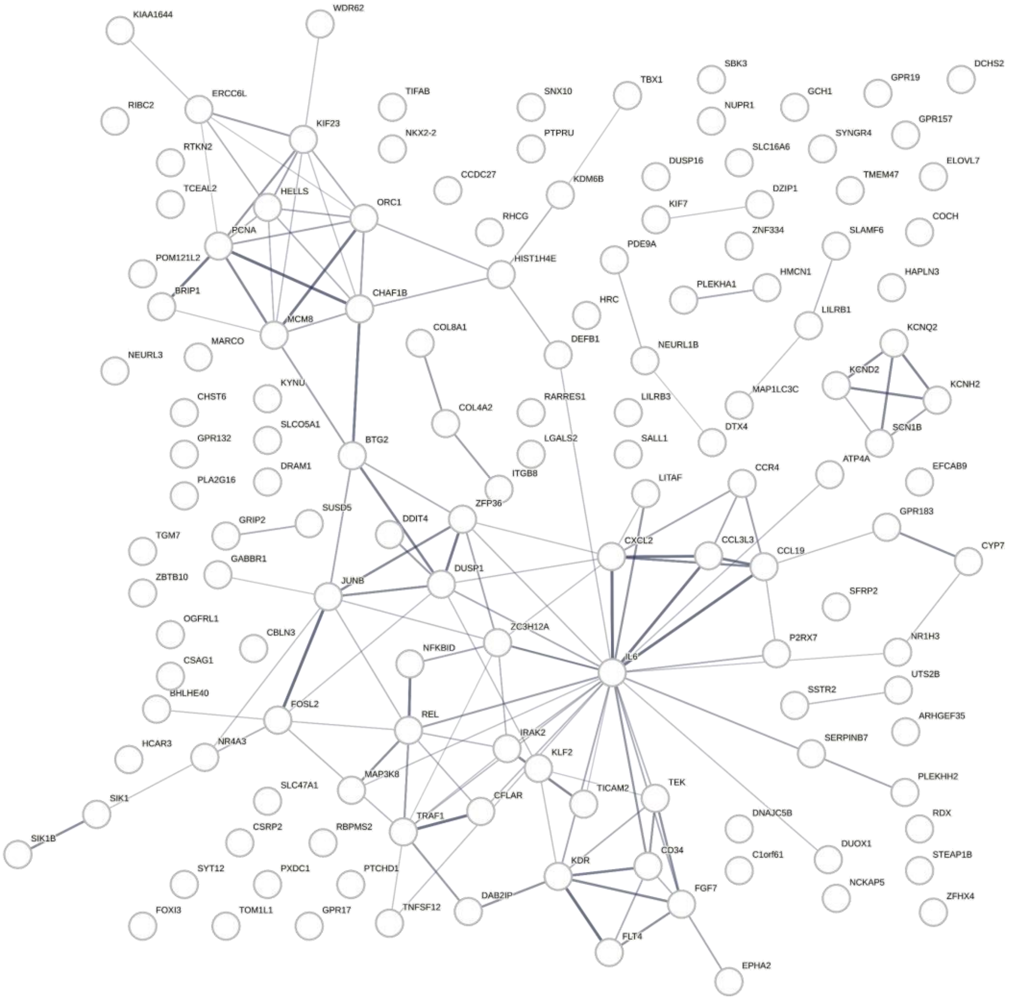
KIAA1644
WDR62
SBK3
DCHS2
TBX1
GPR19
RIBC2
ERCC6L
TIFAB
SNX10
GCH1
NUPR1
KIF23
NKX2-2
PTPRU
SYNGR4
GPR157
RTKN2
SLC16A6
DUSP16
ELOVL7
CCDC27
KDM6B
TMEM47
TCEAL2
HELLS
DZIP1
ORC1
KIF7
RHCG
COCH
PCNA
ZNF334
SLAMF6
PDE9A
POM121L2
HIST1H4E
HMCN1
HAPLN3
BRIP1
CHAF1B
PLEKHA1
HRC
LILRB1
MCM8
COL8A1
KCNQ2
MARCO
DEFB1
NEURL1B
NEURL3
KCND2
KYNU
MAP1LC3C
KCNH2
CHST6
RARRES1
LILRB3
COL4A2
SLCO5A1
DTX4
SCN1B
GPR132
LGALS2
SALL1
BTG2
DRAM1
ATP4A
EFCAB9
PLA2G16
ITGB8
CCR4
LITAF
SUSD5
ZFP36
GPR183
GRIP2
DDIT4
TGM7
CXCL2
CCL3L3
CCL19
CYP7
GABBR1
DUSP1
ZBTB10
JUNB
SFRP2
OGFRL1
ZC3H12A
P2RX7
NR1H3
CBLN3
NFKBID
IL6
CSAG1
UTS2B
SSTR2
BHLHE40
FOSL2
REL
ARHGEF35
SERPINB7
HCAR3
NR4A3
IRAK2
KLF2
MAP3K8
PLEKHH2
SLC47A1
TICAM2
TEK
SIK1
CFLAR
DNAJC5B
RDX
TRAF1
CSRP2
RBPMS2
SIK1B
CD34
DUOX1
C1orf61
STEAP1B
KDR
SYT12
PXDC1
PTCHD1
FGF7
NCKAP5
FOXI3
TOM1L1
GPR17
TNFSF12
DAB2IP
ZFHX4
FLT4
EPHA2

Figure S8

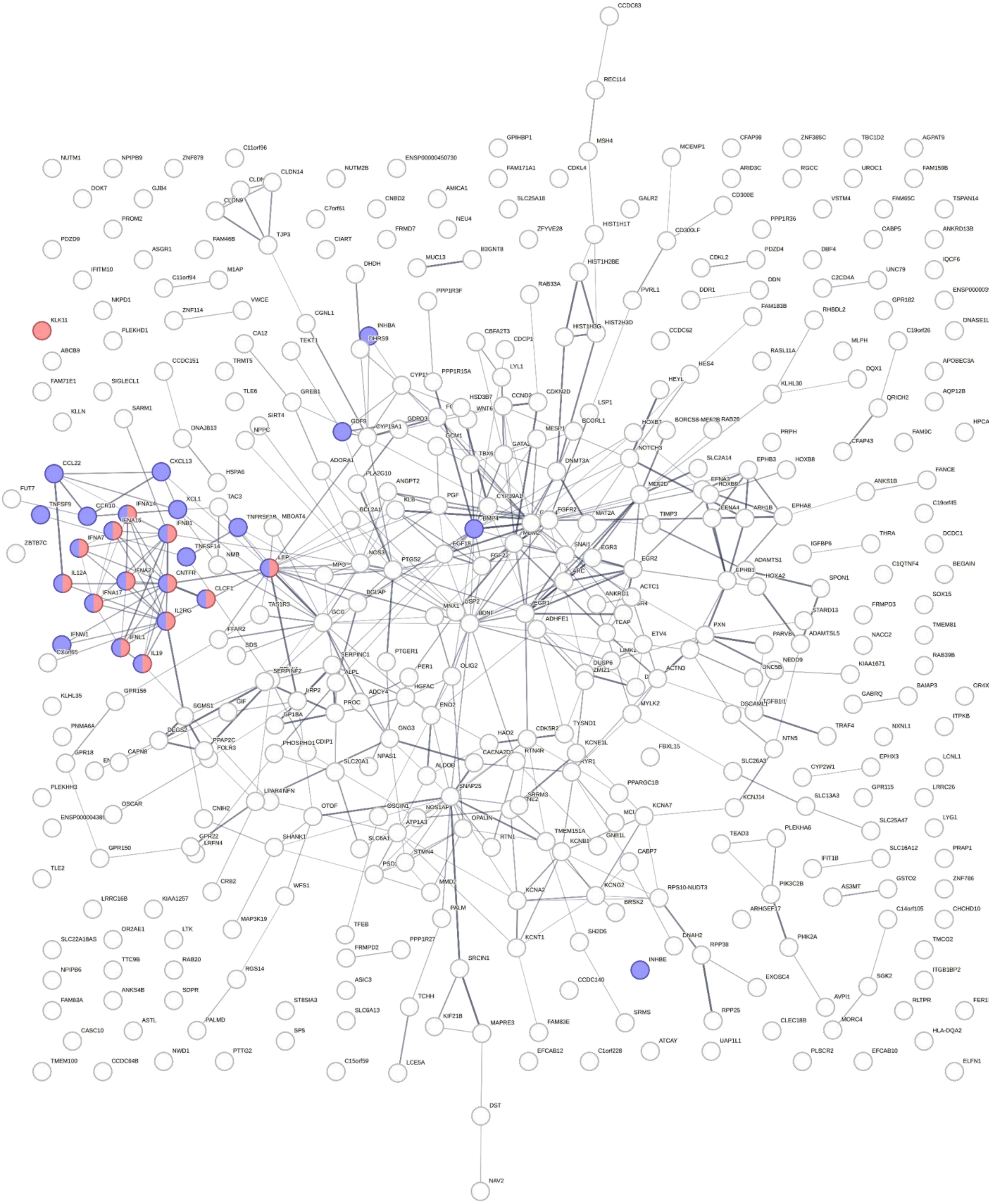
CCDC83
REC114
GPIHBP1
MSH4
MCEMP1
CFAP99
ZNF385C
TBC1D2
AGPAT9
NUTM1
NPIPB9
ZNF878
C11orf96
ENSP00000450730
FAM171A1
CDKL4
ARID3C
RGCC
UROC1
FAM159B
CLDN14
NUTM2B
DOK7
GJB4
CLDN9
CNBD2
AMICA1
SLC25A18
GALR2
CD300E
VSTM4
FAM65C
TSPAN14
C7orf61
PROM2
NEU4
FAM46B
TJP3
FRMD7
ZFYVE28
HIST1H1T
CD300LF
PPP1R36
CABP5
ANKRD13B
PDZD9
CIART
ASGR1
B3GNT8
MUC13
CDKL2
PDZD4
DBF4
IQCF6
IFITM10
C11orf94
M1AP
DHDH
HIST1H2BE
C2CD4A
UNC79
PVRL1
DDR1
DDN
RAB33A
PPP1R3F
NKPD1
VWCE
GPR182
ZNF114
CGNL1
HIST1H3G
HIST2H3D
FAM183B
RHBDL2
KLK11
INHBA
DHRS9
CBFA2T3
CCDC62
DNASE1L
PLEKHD1
CA12
CDCP1
C19orf26
ABCB9
TEKT1
HES4
RASL11A
MLPH
CCDC151
TRMT5
LYL1
DQX1
APOBEC3A
FAM71E1
SIGLECL1
PPP1R15A
CYP11
HEYL
KLHL30
GREB1
CCND2
CDKN2D
TLE6
HSD3B7
WNT6
QRICH2
AQP12B
KLLN
SARM1
LSP1
SIRT4
GDF9
GDRD3
BCORL1
BORCS8-MEF2B
RAB26
DNAJB13
CYP19A1
MESP1
HOXB7
NPPC
FAM9C
HPCA
PRPH
GCM1
NOTCH3
CFAP43
TBX6
GATA
CCL22
CXCL13
ADORA1
DNMT3A
SLC2A14
EPHB3
HOXB8
HSPA6
PLA2G10
FANCE
FUT7
ANGPT2
MEF2D
ANKS1B
XCL1
TAC3
EFNA3
TNFSF9
CCR10
IFNA14
KL8
PGF
CYP39A1
HOXB9
C19orf45
IFNA16
IFNB1
TNFRSF1B
MBOAT4
BCL2A1
EFNA4
EPHA8
BMP4
FGFR2
MAT2A
TIMP3
ZBTB7C
IFNA7
DCDC1
TNFSF14
FGF18
SNAI1
THRA
NMB
NOS3
IGFBP6
LEP
PTGS2
FGF22
EGR3
ADAMTS1
C1QTNF4
IL12A
CNTFR
MPO
EGR2
EPHB1
BEGAIN
IFNA21
ARC
HOXA2
SPON1
IFNA17
CLCF1
BGLAP
ANKRD1
ACTC1
SOX15
MNX1
TAS1R3
FRMPD3
IL2RG
GCG
USP2
BDNF
EGR1
STARD13
IFNW1
FFAR2
TCAP
TMEM81
ADHFE1
ETV4
PXN
IFNL1
PARVB
ADAMTSL5
NACC2
CXorf65
SDS
SERPINC1
PTGER1
LIMK1
DUSP6
NEDD9
RAB39B
IL19
ZMIZ1
UNC5B
KIAA1671
PER1
OLIG2
SERPINF2
ALPL
ACTN3
BAIAP3
KLHL35
GPR156
LRP2
ADCY4
HGFAC
GABRQ
OR4X
SGMS1
GIF
PROC
MYLK2
DSCAML1
TGFB1I1
PNMA6A
ENO2
TYSND1
ITPKB
GP1BA
GNG3
TRAF4
NXNL1
DEGS2
PPAP2C
HAD2
CDK5R2
GPR18
FOLR3
PHOSPHO1
CDIP1
KCNE1L
FBXL15
NTN5
CAPN8
CACNA2D2
RTN4R
EPHX3
LCNL1
NPAS1
SLC20A1
RYR1
SLC26A3
CYP2W1
ALDOB
PLEKHH3
SNAP25
PPARGC1B
GPR115
LRRC26
LPAR4
NFN
KCNJ14
OSCAR
SRRM3
SLC13A3
CNIH2
OTOF
OSGIN1
NOS1AP
KCNA7
ENSP00000438
OPALIN
SLC25A47
LYG1
ATP1A3
TMEM151A
TEAD3
PLEKHA6
GPR22
LRFN4
SHANK1
SLC6A1
RTN1
GNB1L
KCNB1
PRAP1
GPR150
STMN4
CABP7
IFIT1B
SLC16A12
TLE2
CRB2
WFS1
MMD2
KCNA2
KCNG2
PIK3C2B
ZNF786
LRRC16B
KIAA1257
PALM
RPS10-NUDT3
GSTO2
AS3MT
BRSK2
ARHGEF17
C14orf105
CHCHD10
OR2AE1
LTK
MAP3K19
TFEB
SLC22A18AS
KCNT1
SH2D5
DNAH2
PI4K2A
RPP38
TMCO2
TTC9B
RAB20
FRMPD2
PPP1R27
NPIPB6
RGS14
SRCIN1
INHBE
ITGB1BP2
ASIC3
CCDC140
EXOSC4
SGK2
FAM83A
ANKS4B
SDPR
TCHH
ST8SIA3
AVPI1
RLTPR
FER1
SLC6A13
KIF21B
SRMS
RPP25
ASTL
PALMD
MAPRE3
FAM83E
CLEC18B
MORC4
CASC10
SP5
HLA-DQA2
ATCAY
UAP1L1
NWD1
PTTG2
EFCAB12
C1orf228
PLSCR2
EFCAB10
TMEM100
CCDC64B
C15orf59
LCE5A
ELFN1
DST
NAV2

Figure S9

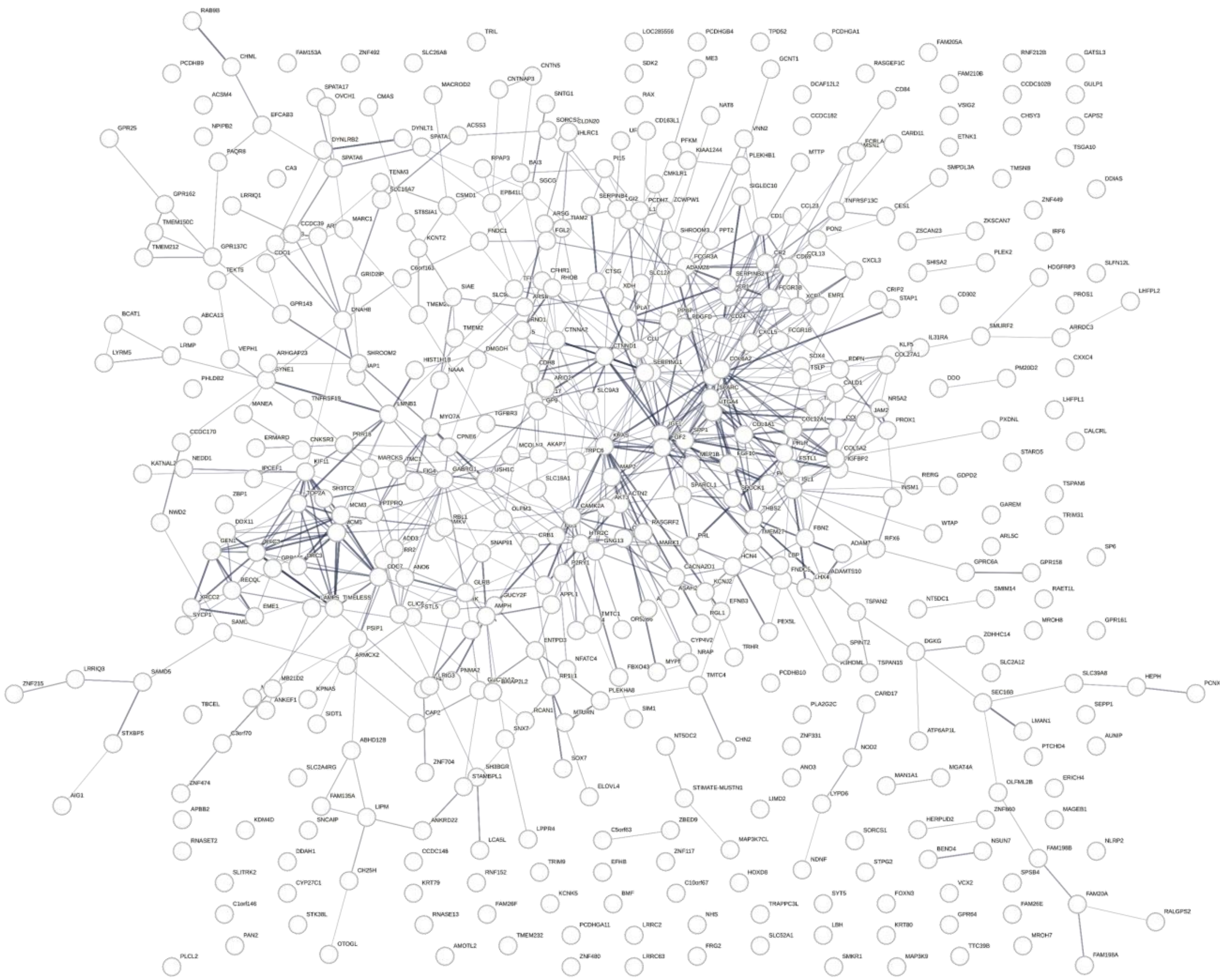

Supplement: Supplementary file 1 [file cells-12-02707-s001.zip › cells-2688738-supplementary.pdf]
